# Reduced efficacy of fluazinam against *Phytophthora infestans* in the Netherlands

**DOI:** 10.1007/s10658-018-1430-y

**Published:** 2018-02-14

**Authors:** H. T. A. M. Schepers, G. J. T. Kessel, F. Lucca, M. G. Förch, G. B. M. van den Bosch, C. G. Topper, A. Evenhuis

**Affiliations:** 1 Wageningen University & Research, PO Box 430, 8200 AK Lelystad, the Netherlands; 2 Wageningen University & Research, PO Box 16, 6700 AA Wageningen, the Netherlands; 3 Potato Research Group, National Agricultural Technology Institute (INTA), P.O. Box 276, 7620 Balcarce, Argentina

**Keywords:** AUDPC, Clonal lineage, Fungicides, Late blight, Potato, Shirlan, Control strategy

## Abstract

*Phytophthora infestans* is the causal organism of potato late blight, the most important disease in potato, the second most important arable crop in Europe. The *P. infestans* population in Europe is well known for its sudden changes in composition. Currently it is composed of a wide variety of genotypes, some of which are dominant clonal lines while others are rare or even unique to a year or location. Fungicides play a crucial role in the integrated control of late blight. Since its introduction in the Netherlands in 1992, fluazinam has been used in late blight control strategies in ware and starch potatoes. It has a broad spectrum of activity and is effective against a range of diseases including potato late blight. Fluazinam interrupts the pathogen cell’s energy production process by an uncoupling effect on oxidative phosphorylation. It is considered to have a low resistance risk. Until recently, reduced efficacy against fluazinam was not detected in *P. infestans* surveys in Europe. In this paper we present the finding of a new clonal lineage (EU_33_A2) of *P. infestans* in the Netherlands and the reduced efficacy of fluazinam to control one of the EU_33_A2 isolates in field experiments carried out in 2011 and 2015 under high disease pressure. The potential effects of this finding on practical late blight control strategies are discussed.

## Introduction

The oomycete *Phytophthora infestans* is the causal organism of potato late blight, the most important disease in potato, the second most important arable crop in Europe. In the Netherlands, potato is cultivated on an area of around 165,000 ha representing an average annual value of about 790 M€ (Haverkort et al. 2008). The *P. infestans* population in the Netherlands has been well known for its sudden changes in composition and is now composed of a wide variety of genotypes, some of which are dominant while others are rare or even unique to a year or location. Since 2004, the Dutch *P. infestans* population is dominated by a clonal lineage called EU_13_A2 (Blue 13) (Cooke et al. 2012; van den Bosch et al. 2012). Other clonal lineages such as EU_1_A1 and EU_6_A1 (Pink 6) have been present in the population for over a decade but never became dominant.

Fungicides play a crucial role in the integrated control strategy for potato late blight deployed in the Netherlands. The average number of sprays per season varies from 7 to 20 depending on the weather, disease pressure and crop (Schepers et al. 2009; Cooke et al. 2011). Since its introduction in the Netherlands in 1992, fluazinam has been used in late blight control strategies in ware and starch potatoes. It was not used in seed potatoes because fluazinam mixed with mineral oil (used to prevent virus transmission) results in phytotoxicity. Because of its excellent preventive and tuber protectant properties, many growers intensively used fluazinam during the entire growing season for many years. Fluazinam is a protective fungicide belonging to the chemical group of the 2,6-dinitroanilines. It has a broad spectrum of activity and is effective against a range of pathogens including *P. infestans* (Anema and Bouwman 1992; Komyoji et al. 1995). In both true fungi and pseudofungi, fluazinam interrupts the fungal cell’s energy production process by an uncoupling effect on oxidative phosphorylation. Its mechanism of action seems to be a simple protonophoric cycle involving protonation/deprotonation of the amino group (Guo et al. 1991). It has been proposed that, as its action is non-specific, selection of resistant strains is extremely unlikely (Tucker et al. 1994). In surveys in Europe, no resistance or reduced sensitivity of *P. infestans* isolates against fluazinam was detected prior to this report (Cooke et al. 1998; Räder and Gisi 2010; Schulte 2011). Also *P. infestans* mutants, resistant to a range of other fungicide active ingredients, did not display resistance or reduced sensitivity to fluazinam (Ziogas et al. 2006). The only case of a reduced efficacy of fluazinam caused by less sensitive isolates was reported in Japan, about 6 years after the introduction of fluazinam, in the control of *Botrytis cinerea* in beans (Tamura et al. 2000; Leroux 2007).

In this paper we present the discovery of Dutch *P. infestans* isolates, belonging to the new *P. infestans* genotype EU_33_A2, displaying a reduced sensitivity to fluazinam in two field trials under high disease pressure and in an in-vitro fungicide sensitivity assay. We hypothesize that the efficacy of fluazinam to inhibit isolates of *P. infestans* clonal lineages regarding zoospore motility provides a good indication for the efficacy of fluazinam under field conditions.

## Materials and methods

### *P. infestans* sample collection and isolate characterization

In the Netherlands *P. infestans* samples have been routinely collected since the 1980’s. From all these samples, *P. infestans* isolates are produced as described by Flier et al. (2001) and stored in liquid nitrogen for later characterization. In addition to these routinely collected samples, in 2010 samples were also taken in fungicide field trials in Lelystad, the Netherlands, in which “fluazinam-treated” plots displayed an unexpected high level of infection. When in August 2011 it became clear that isolates from a new clonal lineage might have a reduced sensitivity to fluazinam, 74 *P. infestans* isolates were collected from late blight-infected plant samples from commercial crops in all potato regions in the Netherlands. SSR genotyping was carried out using the EuroBlight standardized *P. infestans* 12-plex SSR set (Li et al. 2013). The mating type of the isolates was determined using in vitro-crosses with reference isolates of known mating types (Flier et al. 2001). SSR data analysis was done using GeneMapper 3.7. *P. infestans* isolates VK1.4, 80,029, 90,128, ipo-complex, T30–4, 88,133, 98,014 and 428–2 were used as reference isolates originating from various periods in recent Dutch potato late blight history. Also one EU_13_A2 isolate from 2007 and one EU_33_A2 isolate from 2010 were included as reference isolates. Inoculum for field and laboratory experiments was produced from isolates stored in liquid nitrogen as described by Flier et al. (2007).

### An in-vitro assay for sensitivity of *P. infestans* isolates to fluazinam

Twenty *P. infestans* isolates, collected during 2007–2014 and originating from commercial potato crops (Table 1), were tested for their sensitivity to fluazinam using an in-vitro assay. The fluazinam sensitivity assay was modified from the method described by Cooke et al. (1998). Sporangial suspensions (10^5^ sporangia/ml) were prepared from infected leaflets and incubated at 4 °C for 3 h to stimulate zoospore release. Serial dilutions of fluazinam were prepared from the commercial product Shirlan (Syngenta: 500 g/l fluazinam). Aliquots of 250 μl of each fluazinam dilution were pipetted into 24-well plates (Cellstar, Cat.-No.662160). Subsequently, 250 μl aliquots of a sporangial suspension was added to each well to give final concentrations of 10, 1, 0.2, 0.1, 0.05 and 0 μg of fluazinam/ml. Two replicate wells were used per isolate and fluazinam concentration. These 24-well plates were then further incubated at 4 °C before quantifying zoospore motility after 1 and 2 h continued incubation. Zoospore motility was assessed on a 1–3 scale where 1 = not motile, 2 = motile, 3 = very motile. Results were expressed in terms of the minimum inhibitory concentration (MIC), defined as the lowest concentration which completely inhibited zoospore motility (modified from Andrews 2002). This experiment was repeated three times.

**Table 1:** *P. infestans* isolates tested for sensitivity to fluazinam

*P. infestans* isolate	Town of origin	Province of origin	SSR
NL07041	Emmen	Drenthe	EU_13_A2
NL14431	Wessem	Limburg	EU_13_A2
NL11064	Valthermond	Drenthe	EU_13_A2
NL14124	Lelystad	Flevoland	EU_13_A2
NL11147	Lelystad	Flevoland	EU_13_A2
NL10328	Lelystad	Flevoland	EU_33_A2
NL11399	Middenmeer	Noord-Holland	EU_33_A2
NL11410	Renkum	Gelderland	EU_33_A2
NL12082	Reijmerstok	Limburg	EU_33_A2
NL12164	Lelystad	Flevoland	EU_33_A2
NL08277	Valthermond	Drenthe	EU_6_A1
NL07045	Tuil	Gelderland	EU_6_A1
NL11179	Oploo	Noord-Brabant	EU_6_A1
NL14152	Wageningen	Gelderland	EU_6_A1
NL14137	Lelystad	Flevoland	EU_6_A1
NL14296	Lelystad	Flevoland	EU_37_A2^a^
NL14298	Lelystad	Flevoland	EU_37_A2^a^
NL14022	Lelystad	Flevoland	EU_37_A2^a^
NL14033	Middenmeer	Noord-Holland	Clone 1^b^
NL14031	Kreil	Noord-Holland	Clone 1^b^

The isolates, grouped by their SSR genotype, were obtained from potato crops in the Netherlands between 2007 and 2014. The first two digits of the isolate code indicate the year of isolation

^a^EU_37_A2 collected for the first time in 2013

^b^Clone 1 colleted for the first time in 2014

### Field trials

Field trials were carried out in Lelystad, the Netherlands, in 2011 and 2015. The 2011 field trial aimed to assess the efficacy of commonly used potato late blight fungicides to control *P. infestans* isolates NL07041 (EU_13_A2 clonal lineage, Blue 13) and NL10328 (EU_33_A2 clonal lineage, Green 33) (Table 1) under field conditions. The fungicide treatments in the trial (Table 2) were laid out in a randomized block design in four replicates. Plot size was 5.25 m × 11 m. The two isolates were assigned in a split plot design randomly allotted to the replicates. The fungicides were randomly allotted within the blocks for both clonal lineage inoculations. The potato cultivar Maritiema was planted on 30 May 2011 at a density of 4 × 10^4^ plants/ha. Fungicide treatments were applied in a water volume of 250 l/ha using a SOSEF field sprayer with Airmix Flat Fan 110.04 nozzles approximately 50 cm above the foliage. During emergence and fast growth the entire trial was protected from late blight infection by mandipropamid (250 g/l, 0.6 l/ha) cover sprays on 30 June, 7 and 15 July. Designated treatments (Table 2) were subsequently applied in a stable canopy phase on 21 and 26 July 2011. On 2 August, seven days after the last designated treatment on 26 July 2011, the entire field trial was inoculated, using a knapsack sprayer, with *P. infestans* sporangial suspensions (10.000 sporangia/ml) of both isolates individually. Blocks 1 and 2 were inoculated using isolate NL07041 (EU_13_A2) whereas blocks 3 and 4 were inoculated using isolate NL10328 (EU_33_A2). Between blocks 1 & 2 and blocks 3 & 4 a 3 m gap of bare soil was present. Two days after inoculation, the designated fungicide treatments (Table 2) were resumed with applications on 4, 12, 18 and 25 August. The crop was desiccated on 1 September using 4 l/ha diquat-dibromide (374 g/l).

**Table 2:** Treatments in the field experiment in Lelystad, the Netherlands in 2011 to assess the efficacy of fungicides to control the *P. infestans* isolates NL07041 (Blue 13) and NL10328 (Green 33)

Product	Treatment	Dose rate
–	Untreated Control	–
Shirlan Gold	fluazinam (500 g/l)	0.4 l/ha
Shirlan Gold	fluazinam (500 g/l)	0.3 l/ha
Infinito	fluopicolide (62.5 g/l) + propamocarb-HCl (625 g/l)	1.2 l/ha
Revus	mandipropamid (250 g/l)	0.6 l/ha
Ranman A + Ranman B	cyazofamid (400 g/l) + heptamethyltrisiloxane (845,9 g/l)	0.2 + 0.15 l/ha
Curzate M	cymoxanil (4.5%) + mancozeb (68%)	2.5 kg/ha
Valbon	benthiavalicarb(17.5%) + mancozeb (70%)	2.0 kg/ha
Orvego	ametoctradin (300 g/l) + dimethomorph (225 g/l)	0.8 l/ha

The fungicides were sprayed on 21 and 26 July and 4, 12, 18 and 25 August 2011

The 2015 field trial was more focussed on fluazinam-containing products (five products) furthermore including one non-fluazinam tank mix of two products and an untreated control. Also this trial aimed to assess the development of two *P. infestans* isolates (EU_13_A2 and EU_33_A2 clonal lineages, respectively) as influenced by the selection pressure exerted by different fungicide treatments. The trial was designed as a randomized block experiment with 6 treatments (Table 3) and 4 replicates (blocks). Potato cultivar Maritiema was planted on 26 May 2015 at 4 × 10^4^ plants/ha. Spreader rows of unsprayed potato plants of cv. Maritiema were planted in between the blocks. Fungicide treatments were applied in a water volume of 250 l/ha using a CHD-field sprayer with Airmix Flat Fan110.04 nozzles approximately 50 cm above the foliage. During emergence and fast growth the entire trial was protected from late blight infection by mandipropamid (250 g/l, 0.6 l/ha) cover sprays on 19 and 25 June, 2, 10 and 16 July. Artificial inoculation with *P. infestans* was carried out on 16 July 2015. Two plants in untreated spreader-rows next to each plot were inoculated by spraying them each with 8 ml of a sporangial suspension of 10.000 sporangia/ml. One plant was inoculated with NL10328 (EU_33_A2 clonal lineage) while the other plant was inoculated with NL07041 (EU_13_A2 clonal lineage). The designated fungicide treatments (Table 3) were then applied in a stable canopy phase on 23 and 30 July, 6, 13 and 19 August 2015. The first designated treatment was carried out mainly preventively, at that time only a few lesions (0.01% severity) were present in the plots. The crop was desiccated on 2 and 9 September using 4.0 l/ha diquat-dibromide (374 g/l).

**Table 3:** Treatments in the field experiment in Lelystad, the Netherlands in 2015 to assess the development of NL07041 (Blue 13) and NL10328 (Green 33) *P. infestans* isolates under the influence of different spray strategies

Product	Treatment	Dose rate	fluazinam kg/ha
–	Untreated Control	–	–
Shirlan Gold	fluazinam 500 g/l	0.4 l/ha	0.2
Canvas + Dithane DG NT	amisulbrom 200 g/l + mancozeb 75% (tankmix)	0.3 l/ha + 1.75 kg/ha	0
Banjo Forte	fluazinam 200 g/l + dimethomorph 200 g/l	1.0 l/ha	0.2
Kunshi	fluazinam 375 g/l + cymoxanil 250 g/kg	0.5 kg/ha	0.178
Vendetta	fluazinam 375 g/l + azoxystrobin 150 g/l	0.5 l/ha	0.178
Shirlan Gold + Canvas	fluazinam 500 g/l + amisulbrom 200 g/l (tankmix)	0.4 l/ha + 0.3 l/ha	0.2

The fungicides were sprayed on 23 and 30 July, 6, 13 and 19 August 2015

### Quantification of selection pressure in the 2011 and 2015 field trials

To allow for quantification of potential selection pressure exerted by the fungicides applied, *P. infestans* lesions were sampled from the field trials on 18 August 2011 and on 11, 18 and 24 August 2015 in the center rows of the plots. Four lesions were sampled per plot in 2011 and eight lesions were sampled per plot in 2015 and imprinted on Whatman FTA® cards. These samples were subjected to SSR genotyping using the EuroBlight standardized *P. infestans* 12-plex SSR set (Li et al. 2013).

### Disease assessment and data analysis

During the 2011 and 2015 growing seasons potato late blight severity (percentage foliage destroyed by *P. infestans*) was assessed at weekly intervals. To allow for statistical analysis, the standardized area under the disease progress curve (stAUDPC) (Campbell and Madden 1990) was calculated. Analysis of variance (Genstat 18th edition) was performed on severity observations on selected observation dates and on the calculated stAUDPC values. Individual disease ratings were logit transformed. StAUDPC values were not transformed. Comparison of means was carried out using the Fisher’s protected least significant test.

## Results

### *P. infestans* sample collection and isolate characterization

Eight isolates collected in 2010 in fluazinam-treated plots showing an unexpected high level of infection from a field trial in Lelystad, the Netherlands, all belonged to a new SSR clonal lineage of the A2 mating type named EU_33_A2 (Green 33).

**Fig. 1:**
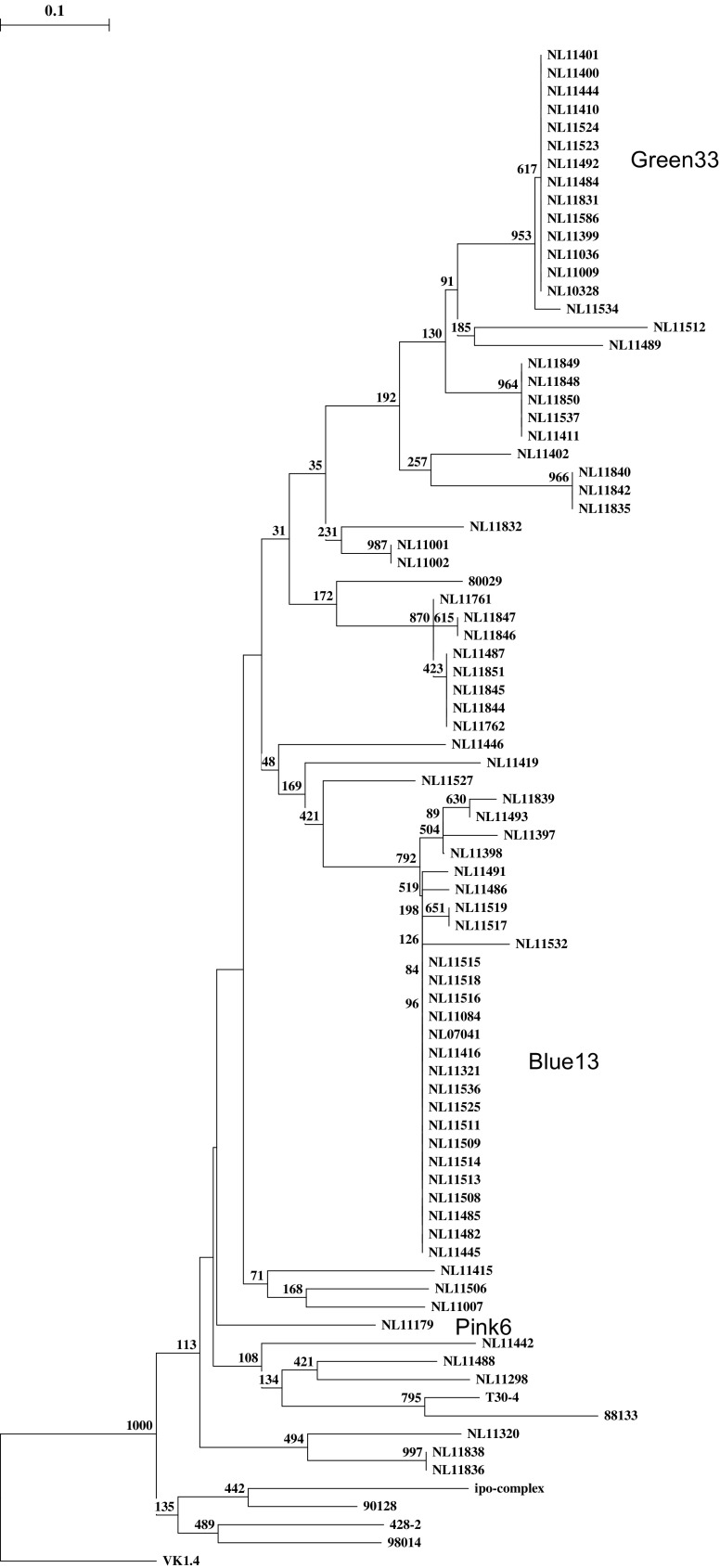
Dendrogram of 74 *P. infestans *isolates collected in the Netherlands in 2011 and 10 reference isolates. The isolates with the EU_33_A2 (Green 33), EU_13_A2 (Blue 13) and EU_6_A1 (Pink 6) genotype are indicated. Bootstrap values to support the knots are given

In 2011, 74 *P. infestans* isolates were successfully isolated from infected plant samples from all potato regions in the Netherlands. Sixty-five isolates originated from infected production crops, 8 isolates came from allotment gardens and one isolate originated from an infected potato dump. SSR genotyping and subsequent data analysis using GeneMapper 3.7 resulted in the dendrogram given in Fig. 1. Thirty-nine SSR genotypes were detected among the eighty-four isolates analysed, including 14 EU_33_A2 isolates, 17 EU_13_A2 isolates and 1 EU_6_A1 isolate. Also two smaller, new, unnamed, groups were found illustrating the continuous flux within the Dutch *P. infestans* population.

**Fig. 2:**
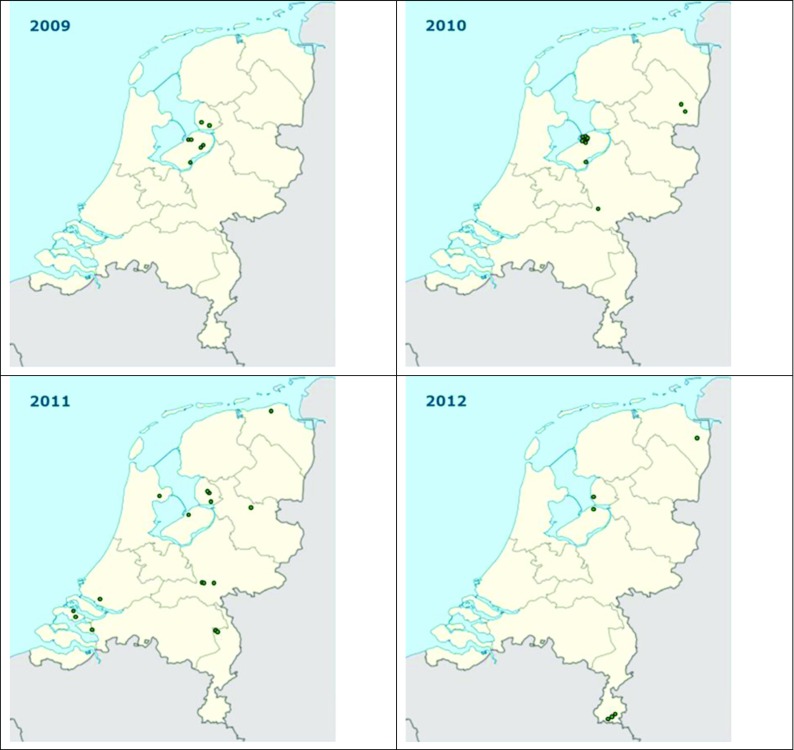
Origins of *P. infestans *isolates with the EU_33_A2 genotype in the years 2009 – 2012. Samples were taken as part of the routine Dutch *P. infestans *monitoring in commercial potato crops. During 2009 – 2012, EU_33_A2 constituted 6%, 14%, 22% and 6% to the Dutch *P. infestans *population. EU_33_A2 was not found before 2009 and after 2012 in the Netherlands

In retrospect, the EU_33_A2 genotype was first found in the province of Flevoland, the Netherlands, in 2009. Seven EU_33_A2 isolates were collected in 2009 in the Netherlands among a total of 110 samples. In 2010, 28 EU_33_A2 isolates were found in the Dutch provinces of Flevoland, Gelderland and Drenthe among a total of 199 samples. In 2011, 14 EU_33_A2 samples, originating from all over the Netherlands, were found in a total of 74 samples. In 2012, 6 isolates were collected belonging to clonal lineage EU_33_A2 of a total of 109 samples. The dynamics of *P. infestans* EU_33_A2 in the Netherlands from 2009 to 2012 as described above are depicted in Fig. 2. EU_33_A2 has not been found in the Netherlands in agricultural practice since 2013 up to 2016.

### In-vitro sensitivity of *P. infestans* isolates to fluazinam

A collection of 20 *P. infestans* isolates, obtained from commercial potato crops in the Netherlands between 2007 and 2014 and representing 5 clonal lineages, were tested for sensitivity to fluazinam in a replicated in-vitro assay. Analysis of variance of the resulting MIC values (Table 4) demonstrated that isolates having a EU_33_A2 or EU_37_A2 SSR genotype had significantly higher MIC values when compared to isolates with a EU_13_A2, EU_6_A1 or Clone 1 genotype. These differences were present after 1 and after 2 h of incubation of zoospores in their respective fluazinam concentrations.

**Table 4:** Sensitivity to fluazinam in the replicated zoospore motility assay for *P. infestans* isolates belonging to five clonal lineages collected in the Netherlands from 2007 to 2014

	No. of isolates tested	MIC value (μg/ml)	
		Incubation time 1 h	Incubation time 2 h
EU_13_A2 (Blue 13)	5	0.2 a^a^	0.2 a
EU_33_A2 (Green 33)	5	9.9 b	6.9 c
EU_6_A1 (Pink 6)	5	0.8 a	0.6 a
EU_37_A2 (Dark Green 37)	3	9.0 b	4.0 b
Clone 1	2	1.0 a	1.0 a

^a^Within columns values followed by the same letter are not significantly different according to Fisher’s protected least significant difference test at *P* = 0.05

### Field trials in 2011 and 2015

In the 2011 field trial all potato plants in blocks 1 and 2 were inoculated with *P. infestans* NL07041 (EU_13_A2) and those in blocks 3 and 4 with NL10328 (EU_33_A2). Results are given in Tables 5 and 6.

**Table 5:** Foliar blight severity after application of the different treatments, assessed at weekly intervals, in the field trial in Lelystad, the Netherlands in 2011, and mean stAUDPC-values

Treatment	Isolate^a^	Severity (%)					stAUDPC (%)
		4 / 8	11 / 8	19 / 8	26 / 8		
Untreated control	Blue 13	8.8	75.0	–	–	–	
	Green 33	12.5	87.5	–	–	–	
fluazinam (0.4 l/ha)^c^	Blue 13	1.5	5.0	12.5	77.5	23.2	...de.^b^
	Green 33	1.8	20.0	80.0	99.0	45.8	.....f
fluazinam (0.3 l/ha)	Blue 13	1.3	6.3	12.5	80.0	23.0	...de.
	Green 33	1.8	22.5	85.0	99.0	48.4	.....f
fluopicolide + propamocarb	Blue 13	0.1	1.8	3.5	2.0	2.1	a.....
	Green 33	0.2	1.3	2.0	1.5	1.4	a.....
mandipropamid	Blue 13	1.5	4.0	12.5	42.5	14.0	.bcd..
	Green 33	1.0	2.3	4.3	20.0	8.4	abc...
cyazofamid	Blue 13	2.0	2.0	4.5	13.8	6.1	ab....
	Green 33	2.5	1.5	3.5	17.5	7.3	abc...
mancozeb + cymoxanil	Blue 13	1.8	4.8	13.8	50.0	16.7	..cd..
	Green 33	2.0	2.3	6.3	25.0	9.0	abc...
mancozeb + benthiavalicarb	Blue 13	2.5	3.0	7.5	30.0	10.5	abc...
	Green 33	0.7	1.0	1.3	3.8	2.0	a.....
ametoctradin + dimethomorph	Blue 13	2.5	7.5	37.5	90.0	32.2	....e.
	Green 33	0.8	3.8	10.0	52.5	17.2	..cd..

^a^Plots in block 1 and 2 were spray inoculated with *P. infestans* isolate NL07041 (EU_13_A2, Blue 13). Plots in blocks 3 and 4 were spray inoculated with *P. infestans* isolate NL10328 (EU_33_A2, Green 33)

^b^stAUDPC values followed by the same letter are not significantly different according to Fisher’s protected least significant difference test at P = 0.05

^c^The test products were sprayed 21 and 26 July; 4, 12, 18 and 25 August

**Table 6:** *P. infestans* genotypic composition under fungicide selection pressure in the 2011 field trial in Lelystad, the Netherlands based on 8 samples per treatment – inoculant combination

Treatment	*P. infestans* SSR genotype detected (%)					
	Block 1+ 2 inoculated with Blue13			Block 3 + 4 inoculated with Green33		
	EU_13_A2 clonal lineage	EU_33_A2 clonal lineage	Other genotypes	EU_13_A2 clonal lineage	EU_33_A2 clonal lineage	Other genotypes
fluazinam (0.4 l/ha)	38 a^a^	12 a	50 a	0 a	100 c	0 a
fluazinam (0.3 l/ha)	80 a	10 a	10 a	10 ab	73 bc	17 ab
fluopicolide + propamocarb-HCl	100 a	0 a	0 a	77 b	0 a	23 abc
mandipropamid	70 a	0 a	30 a	25 ab	25 ab	50 cd
cyazofamid	62 a	0 a	38 a	55 ab	12 a	33 abcd
mancozeb+ cymoxanil	100 a	0 a	0 a	37 ab	25 ab	38 bcd
mancozeb+benthiavalicarb	87 a	0 a	13 a	60 ab	30 ab	10 ab
ametoctradin + dimethomorph	62 a	0 a	38 a	25 ab	10 a	65 d
	EU_13_A2 clonal lineage	EU_33_A2 clonal lineage	Other genotypes	EU_13_A2 clonal lineage	EU_33_A2 clonal lineage	Other genotypes
fluazinam	59 a^a^	11 b	30 a	5 a	87 b	8 a
non-fluazinam	80 a	0 a	20 a	47 b	17 a	36 b

Plots in block 1 and 2 were spray inoculated with *P. infestans* isolate NL07041 (EU_13_A2). Plots in blocks 3 and 4 were spray inoculated with *P. infestans* isolate NL10328 (EU_33_A2) Samples were taken on 18 August 2011. This was 16 days after inoculation of block 1 and 2 with NL07041 (EU_13_A2) and block 3 and 4 with NL10328 (EU_33_A2). At that time the test products were sprayed 4 times. Means in the same column followed by the same letter are not significantly different according to Fisher’s protected least significant test at *P* = 0.05. The upper part of the table gives the genotype frequency for each fungicide separately, the lower table gives the pooled data for fluazinam and non-fluazinam fungicides

^a^Within columns values followed by the same letter are not significantly different according to Fisher’s protected least significant difference test at *P* = 0.05

The late blight epidemic developed very rapidly in the untreated control plots. Severity in these untreated plots ranged from 70% to 90% on 11 August 2011. To try and negate the negative consequences of the extreme disease pressure projected from these plots, they were desiccated immediately following the severity assessment on 11 August 2011. Therefore, stAUDPC values for the untreated control plots could not be calculated.

Epidemic development in the fluazinam-sprayed plots in blocks 3 and 4, inoculated with the EU_33_A2 isolate, was significantly faster when compared to the other fungicides in these blocks. It was also faster than epidemic development in the fluazinam-treated plots in blocks 1 and 2, inoculated with the EU_13_A2 isolate. Also, no significant dose rate effect of fluazinam (0.3 l/ha versus 0.4 l/ha) was found, regardless of the inoculant (Table 5). All other non-fluazinam-treatments resulted in an equivalent or slower epidemic development of the EU_33_A2 isolate versus the EU_13_A2 isolate as reflected in their respective stAUDPC values.

On 18 August 2011, four infected leaflets with a single late blight lesion were collected from each plot and used to establish the SSR genotype of the *P. infestans* responsible. Since the field experiment was inoculated with two isolates (EU_13_A2; EU_33_A2), the resulting SSR genotypes were classified into three groups: EU_13_A2, EU_33_A2 and “other” genotypes. The presence of “other” genotypes is most likely the result of *P. infestans* influx from infected potato fields in the area surrounding the trial. Results are summarized in Table 6 where the relative *P. infestans* population composition is given per treatment and for both inoculants based on 8 samples per treatment-inoculant combination. In the treatments inoculated with NL07041 (EU_13_A2), the *P. infestans* population was composed of EU_13_A2 and “other” genotypes for all treatments except for both fluazinam-treatments. A low-level influx of EU_33_A2 was able to establish itself in fluazinam-treated plots. EU_33_A2 was not able to establish itself in any of the plots treated otherwise.

In the treatments inoculated with NL10328 (EU_33_A2) the *P. infestans* population was composed of EU_13_A2, EU_33_A2 and “other” genotypes except for plots treated with fluopicolide + propamocarb where EU_33_A2 was not found. The effect of the fluazinam-treatments on the *P. infestans* population composition is more pronounced for the 0.4 l/ha dose rate. In these plots *P. infestans* was 100% EU_33_A2 genotype. This effect was less pronounced for the fluazinam 0.3 l/ha treatment but the trend was still visible. All other treatments seemed to result in similar proportions for the EU_13_A2, EU_33_A2 and “other” genotypes.

In the 2015 field trial, the epidemic developed quickly in the untreated spreader rows from the end of July onwards. On 11 August, the disease severity in the untreated control plots was already at 75%. During the second week of August, the epidemic also strongly increased in the treated plots (Table 7). The stAUDPC was significantly lower in all treated plots when compared to the untreated control.

**Table 7:** Foliar blight severity after application of the different treatments assessed at weekly intervals in the field trial in Lelystad, the Netherlands in 2015 and mean stAUDPC

Treatment	Infected foliage (%)								stAUDPC	
	5/8		14/8		21/8		28/8			
Untreated control	(55.0)		(92.3)		(99.0)		(100)		(65.5)	
fluazinam	0.30	b^a^	24.4	d	75.0	c	96.5	e	27.8	d
amisulbrom + mancozeb	0.07	a	3.9	ab	25.0	a	63.8	b	11.8	ab
fluazinam + dimethomorph	0.06	a	5.9	b	56.3	b	82.5	cd	20.1	c
fluazinam + cymoxanil	0.08	a	13.4	c	62.5	bc	93.3	de	24.0	cd
fluazinam + azoxystrobin	0.09	a	2.6	a	21.9	a	72.5	bc	12.9	b
fluazinam + amisulbrom	0.06	a	2.8	a	17.5	a	48.8	a	8.8	a

^a^Values in the same column followed by the same letter are not significantly different according to Fisher’s protected least significant test at P = 0.05

For a more accurate comparison of the sprayed treatments, the untreated control was then excluded from further statistical analysis. The StAUDPC of the fluazinam + amisulbrom treatment was then found significantly lower than all other sprayed treatments except for amisulbrom + mancozeb. The treatments with fluazinam, fluazinam + cymoxanil and fluazinam + dimethomorph resulted in the highest stAUDPC values, significantly higher than stAUDPC values for the other sprayed treatments.

In the 2015 field trial, eight infected leaflets with a single late blight lesion were collected from each plot on three dates: 11, 18 and 24 August, 4, 5 and 6 weeks post inoculation respectively. The resulting *P. infestans* SSR genotypes were again classified into three groups: EU_13_A2, EU_33_A2 and “other” genotypes. Results are summarized in Table 8. Based on averages in the amisulbrom + mancozeb treatment (without fluazinam) a significantly higher percentage of EU_13_A2 genotypes and a significantly lower percentage of EU_33_A2 genotypes was recovered compared to all other -fluazinam-containing treatments. In plots treated with fluazinam and fluazinam + cymoxanil the percentage of EU_33_A2 genotypes was significantly higher and the percentage of EU_13_A2 genotypes was significantly lower when compared to the plots treated with other, fluazinam-containing treatments except for fluazinam + dimethomorph. Comparing fluazinam treated plots with non-fluazinam treated plots, the frequency of EU_33_A2 found in the fluazinam treated plots was significantly higher than EU_13-A2 at each assessment date. In the non-fluazinam treated plots this was vice-versa, i.e. EU_13_A2 was found more frequently than EU_33_A2 at each assessment date.

**Table 8:** *P. infestans* composition in the plots of the field trial in Lelystad, the Netherlands 2015 treated with different fungicides based on 8 samples per treatment

Treatment	*P. infestans* SSR genotype detected (%)											
	EU_13_A2				EU_33_A2				“other” *P. infestans* genotypes			
date	11/8	18/8	24/8	Average	11/8	18/8	24/8	Average	11/8	18/8	24/8	Average
fluazinam^b^	46 a^a^	36 a	13 ab	31 a	51 b	54 b	74 cd	60 c	3 a	10 b	13 a	9 a
amisulbrom + mancozeb	78 b	97 c	93 c	89 c	6 a	3 a	0 a	3 a	16 a	3 a	7 a	7 a
fluazinam + dimethomorph	75 b	41 ab	31 ab	49 ab	22 a	56 b	45 b	41 b	3 a	3 a	24 a	10 a
fluazinam + cymoxanil	45 a	47 ab	3 a	32 a	52 b	53 b	86 d	63 c	3 a	0 a	11 a	5 a
fluazinam + azoxystrobin	63 ab	60 b	35 b	53 b	28 ab	40 b	38 b	36 b	9 a	0 a	27 a	13 a
fluazinam + amisulbrom	45 a	88 c	35 b	56 b	52 b	9 a	51 bc	38 b	3 a	3 a	14 a	7 a
	EU_13_A2				EU_33_A2				“other” *P. infestans* genotypes			
	11/8	18/8	24/8	Average	11/8	18/8	24/8	Average	11/8	18/8	24/8	Average
fluazinam	54 a^a^	54 a	23 a	44 a	41 b	43 b	59 b	47 b	4 a	3 a	18 a	8 a
non-fluazinam	78 b	97 b	93 b	89 b	6 a	3 a	0 a	3 a	16 b	0 a	7 a	7 a

The trial was inoculated on 16 July 2016 in the spreader rows with NL07041 (EU_13_A2) and with NL10328 (EU_33_A2). The upper table gives the genotype frequency for each fungicide separately, the lower table gives the pooled data for fluazinam and non-fluazinam fungicides

^a^Values in the same column followed by the same letter are not significantly different according to Fisher’s protected least significant difference test at P = 0.05

^b^The test products were sprayed on 23 and 30 July; 6, 13 and 19 August

## Discussion

Following the discovery of the new *P. infestans* SSR genotype EU_33_A2 (Green 33), in a field experiment in Lelystad, the Netherlands in 2010 in which fluazinam-treated plots showed an unexpectedly high level of infection, the entire Wageningen Plant Research *P. infestans* collection was checked for this new genotype. As a result, the EU_33_A2 genotype was first found in Lelystad in 2009 and then in many other locations in the Netherlands, other than the trial site in Lelystad, in 2010. These findings led to the 2011 field trial to investigate the efficacy of the most common potato late blight fungicides registered in the Netherlands against this new clonal lineage.

The 2011 field experiment was carried out under high disease pressure using a sub-optimal spray schedule to be able to assess the efficacy of the different fungicides towards controlling two *P. infestans* isolates belonging to the clonal lineages EU_13_A2 (Blue 13) and a EU_33_A2 (Green 33). The efficacy of most fungicides to control potato late blight was comparable for both isolates except for fluazinam. For both isolates however the control efficacy of fluazinam against the EU_33_A2 isolate was significantly lower than for the EU_13_A2 isolate.

In all plots inoculated with the EU_33_A2 isolate, the EU_33_A2 population was reduced in favour of invading EU_13_A2 genotypes, except for plots sprayed with fluazinam. In 2011, plots inoculated with the EU_13_A2 isolate, EU_33_A2 was not found except in the plots sprayed with fluazinam, even though at a low level. In the 2015 trial, EU_33_A2 was found predominantly in fluazinam treated plots and not in the plot treated with the non-fluazinam reference. In non-fluazinam treated plots EU_13_A2 was the common genotype found. These findings strongly suggest a competitive advantage for EU_33_A2 in plots sprayed with fluazinam compared with other *P. infestans* SSR genotypes including EU_13_A2. The relative position of plots relative to the main wind direction, did not account for unidirectional isolate flow between blocks. EU_13_A2 was inoculated onto the two southern blocks and EU_33_A2 onto the northern blocks. Prevailing wind direction in August 2011 varied from southwest to northwest. In August 2011 the wind blew 12 days from a southerly and 13 days from a northerly direction. Alternatively EU_33_A2 might be more sensitive to non-fluazinam active ingredients than EU_13_A2, this might also result in a decrease of EU_33_A2 in the Netherlands. We have not investigated fungicide sensitivity of EU_33_A2 to other active ingredients. Looking at the non-fluazinam treatments no significant difference of disease severity expressed as stAUDPC was found in the field in 2011 between blocks inoculated with EU_13_A2 and EU_33_A2, respectively, except for ametoctradin + dimethomorph (Table 5).

The high disease pressure and sub-optimal spray timing in this field experiment were highly challenging for the purely protectant fluazinam. Some of the other fungicides benefitted from (additional) curative components which helped to achieve a better control under these difficult conditions. However this does not explain the significant difference in control by fluazinam with the EU_13_A2 and EU_33_A2 isolates. Although EU_13_A2 is generally known for its high aggressiveness (Cooke et al. 2012), fluazinam performed better against the EU_13_A2 isolate than the EU_33_A2 isolate. The relatively good levels of control of the EU_33_A2 isolate with the non-fluazinam fungicides indicated that this EU_33_A2 isolate is probably less aggressive than the common EU_13_A2 isolates.

The field trial carried out in 2015 was artificially inoculated with the same EU_33_A2 and EU_13_A2 isolates as the 2011 field trial. There was however a clear difference in the way the artificial inoculation was carried out. In 2011 the sporangial suspensions were applied full-field following two designated fungicide applications. In 2015, individual plants in the untreated spreader rows were inoculated with individual *P. infestans* isolates before the first designated treatments were applied on the plots. In addition, the 2011 trial was designed to test whether the EU_33_A2 isolate could be controlled by the different late blight fungicides on the Dutch market. The 2015 trial was designed to test the lower sensitivity of the EU_33_A2 isolate under field conditions and to investigate the effect of the different fungicides in competition between the EU_13_A2 and EU_33_A2 isolates. From the 2015 trial it was clear that in the absence of fluazinam, the EU_33_A2 isolate was outcompeted by the EU_13_A2 isolate. In commercial fields this phenomenon was also observed when, after the widespread occurrence of EU_33_A2 in Dutch commercial potato fields in 2011, the use of fluazinam dropped dramatically in 2012 and afterwards. This immediately resulted in a much lower frequency of EU_33_A2 in the Dutch *P. infestans* population: 6% in 2012 and 0% in 2013. This is illustrated by the genotype frequency maps on the EuroBlight website (www.euroblight.net). However this does not mean that EU_33_A2 has disappeared in the Netherlands. First of all, the routine *P. infestans* sampling is limited in number of samples that can be collected per growing season. Secondly the reduced sensitivity to fluazinam does not seem to be limited to the EU_33_A2 genotype. From the in-vitro assay (Table 4) it was also clear that the sensitivity to fluazinam of EU_37_A2 isolates, which was found in the Netherlands for the first time in 2013, was clearly reduced.

The selective advantage of the EU_33_A2 isolate in plots sprayed with fluazinam could be caused by a change in sensitivity towards fluazinam in this isolate. Fluazinam is generally very effective in preventing germination of *P. infestans* sporangia and zoospores. Zoospores in particular are very sensitive to low concentrations of fluazinam (Cooke et al. 1998). Despite its broad spectrum of activity and the classification of the Fungicide Resistance Action Committee (www.frac.info) that the risk for development of resistance is low, the intensive use of fluazinam in the Netherlands since 1992 may have exerted sufficient selection for specific genotypes of *P. infestans* with reduced sensitivity. The results of the in-vitro assays of sensitivity of zoospore germination to fluazinam (Table 4) certainly seem to support this hypothesis. In this replicated assay, two *P. infestans* clones, EU_33_A2 and EU_37_A2, were less sensitive to fluazinam. MIC values were approximately 9 times higher for EU_33_A2 and EU_37_A2 when compared clonal lines EU_13_A2, EU_6_A1 and “Clone 1”.

Resistance management to lower the selection pressure towards isolates with a lower fluazinam sensitivity could consist of reducing the number of fluazinam sprays in a growing season and/or applying fluazinam in combination with active ingredients/fungicides with a different mode of action in ready-formulated products or in tank mixes. The results of the 2015 trial show that the combination partners with weak protectant characteristics such as cymoxanil did not reduce the selection pressure towards EU_33_A2 compared to fluazinam solo. Other, better protectant partners did however reduce the selection pressure towards EU_33_A2. Grünwald et al. (2006) investigated the selection for fungicide resistance, within a growing season, in field populations of *P. infestans* in the central highlands of Mexico. Isolates were detected that could grow on artificial media amended with 100 μg/ml fluazinam but no shift in frequency distribution was detected during the course of the growing season. They concluded that given the high potential for gene flow and the fact that strains tolerant to fluazinam were already established, clonal reproduction of these strains under fluazinam selection pressure could lead to a situation analogous to that with metalaxyl. The development of resistance to metalaxyl is however completely different to the occurrence of isolates with a lower sensitivity to fluazinam as described in this paper. Resistance to metalaxyl was already detected 1–2 years after its introduction and has since then been described in many countries worldwide. In commercial crops the isolates with a lower sensitivity to fluazinam have until now only been found in the Netherlands after 30 years of intensive fluazinam use. Another important difference is that metalaxyl-resistant isolates of *P. infestans* are generally at least as fit as the sensitive isolates even in absence of fungicide selection (Gisi and Cohen 1996) whereas there are strong indications that the decreased sensitivity to fluazinam of EU_33_A2 isolates has a fitness penalty. Cooke (1991) hypothesized that after many years of selection by phenylamide fungicides (including metalaxyl), resistance and fitness have probably been combined to produce strains as fit as the wild type. The future will show whether prolonged selection pressure by fluazinam will also result in strains with a decreased sensitivity without a fitness penalty.

The results obtained in the 2011 and 2015 field trials indicate that isolate NL10328 (EU_33_A2) is less sensitive to fluazinam compared to isolate NL07041 (EU_13_A2) and the other genotypes present. Apart from the findings of Grünwald et al. (2006), only one other case of reduced sensitivity of *P. infestans* to fluazinam has been reported. Significantly lower control of *P. infestans* in potato crops by fluazinam was observed in Denmark in artificially inoculated field trials in 2006 and 2007 (Nielsen 2013). *P. infestans* isolates from these trials were tested for sensitivity to fluazinam on artificial media amended with fluazinam but reduced sensitivity was not observed. In these tests mycelial plugs of *P. infestans* isolates were placed on artificial media amended with fluazinam. Since the main effect of fluazinam is on zoospore motility rather than on mycelium growth, it is not surprising that a reduced sensitivity was not observed in agar tests.

Reduced sensitivity to fluazinam was also observed for *B. cinerea* in Japanese bean fields. *B. cinerea* isolates collected from bean fields that had never been treated with fluazinam mainly exhibited EC_50_ values around 0.003 ppm. In similar tests with *B. cinerea* isolates from fluazinam-treated crops, two levels of sensitivity were found. Isolates exhibiting a low resistance factor (about 10 x) or a high resistance factor (about 10,000 x) were detected. Under field conditions, the fluazinam-resistant isolates, especially in a programme involving three treatments with fluazinam, were not effectively controlled (Leroux 2007; Tamura et al. 2000). The biochemical background of fluazinam resistance in *B. cinerea* is not known, but a mechanism involving detoxification was suggested. This assumption was based on the fact that in mammalian mitochondria, fluazinam is probably metabolically detoxified by a glutathione conjugation mechanism. It remains to be determined if such a phenomenon occurs in *B. cinerea*-resistant strains (Leroux et al. 2002).

In surveys in Europe no isolates of *P. infestans* resistant against fluazinam were found prior to this report (Cooke et al. 1998; Räder and Gisi 2010; Schulte 2011; Kessel et al. 2011). Also mutants of *P. infestans* resistant to a range of fungicides did not show a reduced sensitivity to fluazinam (Ziogas et al. 2006). These surveys all used different fungitoxicity tests to determine whether changes in sensitivity had occurred. Cooke et al. (1998) used zoospore motility as the response variable whereas Räder and Gisi (2010) and Schulte (2011) measured the radial development of mycelium on artificial media. Kessel et al. (2011) studied the development of *P. infestans* on discs cut from treated potato leaves. The MIC values for zoospore motility determined by Cooke et al. (1998) ranged from 0.02 to 0.06 μg/ml which is comparable with the MIC values for the EU_13_A2 isolates reported in this study. In the current study, there was a good correlation between the results from the zoospore motility tests and the results obtained from the field trials in 2011 and 2015. A comparison of the zoospore motility test and agar growth test to assess the sensitivity to fluazinam has not been carried out with the same isolates. Further research is needed to determine whether the EU_33_A2 and EU_37_A2 genotypes also show a lower sensitivity in the agar growth test. If these isolates do not show a lower sensitivity to fluazinam in the agar growth test but a clear effect in the zoospore motility test, then the agar growth tests are not useful for assaying reduced sensitivity to fluazinam.

Further *P. infestans* population monitoring and research in the Netherlands, and other countries where fluazinam is used to control *P. infestans* in potatoes, are needed to demonstrate how widespread reduced sensitivity to fluazinam is. Furthermore it is also necessary to know whether reduced fluazinam-sensitivity is found outside the EU_33_A2 clonal lineage -such as in EU_37_A2. For agricultural practise it is eminent to know whether these genotypes are also more difficult to control using fluazinam in commercial fields. In that case the indication of reduced fitness of NL10328 (EU_33_A2 clonal lineage) in absence of fluazinam might be very important for the design of efficient resistance management strategies.
